# A View into Seed Autophagy: From Development to Environmental Responses

**DOI:** 10.3390/plants11233247

**Published:** 2022-11-26

**Authors:** Raquel Iglesias-Fernández, Jesús Vicente-Carbajosa

**Affiliations:** 1 Centro de Biotecnología y Genómica de Plantas-Severo Ochoa (CBGP, UPM-INIA), Universidad Politécnica de Madrid (UPM)—Instituto Nacional de Investigación y Tecnología Agraria y Alimentaria (CSIC/INIA), 28223 Pozuelo de Alarcon, Spain; 2 Departamento de Biotecnología-Biología Vegetal, Escuela Técnica Superior de Ingeniería Agronómica, Alimentaria y de Biosistemas, Universidad Politécnica de Madrid (UPM), 28040 Madrid, Spain

**Keywords:** autophagy, endoplasmic reticulum, environment, plants, seed germination, seed maturation, selective autophagy

## Abstract

Autophagy is a conserved cellular mechanism involved in the degradation and subsequent recycling of cytoplasmic components. It is also described as a catabolic process implicated in the specific degradation of proteins in response to several stimuli. In eukaryotes, the endoplasmic reticulum accumulates an excess of proteins in response to environmental changes, and is the major cellular organelle at the crossroads of stress responses. Return to proteostasis involves the activation of the Unfolded Protein Response (UPR) and eventually autophagy as a feedback mechanism to relieve protein overaccumulation. Recent publications have focused on the relevance of autophagy in two central processes of seed biology: (i) seed storage protein accumulation upon seed maturation and (ii) reserve mobilization during seed imbibition. Although ER-protein accumulation and the subsequent activation of autophagy resemble the Seed Storage Protein (SSP) deposition during seed maturation, the molecular connection between seed development, autophagy, and seed response to abiotic stresses is still an underexplored field. This mini-review presents current advances in autophagy in seeds, highlighting its participation in the normal course of seed development from embryogenesis to germination. Finally, the function of autophagy in response to the seed environment is also considered, as is its involvement in controlling seed dormancy and germination.

## 1. Plant Autophagy: General Background

The word autophagy comes from ancient Greek and means self-eating. In eukaryotic organisms, autophagy is a conserved cellular mechanism involved in the degradation of cytoplasmic constituents to reuse their basic units [1,2]. Autophagy removes any unnecessary, damaged, or dysfunctional cellular components; therefore, it is a primary cell recycling system in eukaryotes. In the plant cell, autophagy occurs in the acidic central vacuole in three different ways: microautophagy, macroautophagy, and mega-autophagy. In microautophagy, the central vacuole engulfs cytoplasmic constituents (i.e., cargos) from the cytosol by direct invagination of the tonoplast [3]. In contrast, macroautophagy involves the sequestration and delivery of cargo by the autophagosome, a double-membrane vesicle that appears to originate from the endoplasmic reticulum (ER). At the central vacuole, the tonoplast fuses with the outer layer of the autophagosome, and the inner membrane with the cargo inside is engulfed. There, vacuolar hydrolases degrade and return the broken-down materials to the cytosol [4,5,6]. How autophagy recognizes its cargo in the plant cell is not well understood. Mega-autophagy is the massive degradation of the cellular components that occurs in the late phase of programmed cell death (PCD) when the central vacuole releases large amounts of hydrolases into the cytoplasm [7]. The autophagic process occurs at basal levels, but intensifies in response to specific developmental programs (e.g., senescence, germination, and reproduction) and to support plant survival under abiotic and biotic stresses [8,9,10,11,12].

The autophagy molecular machinery comprises approximately 40 proteins conserved in plants, animals, and fungi, and some designated AuTophaGy-related (ATGs) proteins. ATGs are heterogeneous in function and belong to over seventeen protein families, such as kinases, ubiquitin-like proteins, Vacuolar Protein Sorting (VPS) factors, and cysteine proteases [13]. In *Arabidopsis thaliana*, ATGs are part of four complexes involved in autophagosome formation: (i) the ATG1 kinase complex that activates autophagy in response to nutrient starvation—the kinase Target Of Rapamycin (TOR) inactivates the ATG1 complex by phosphorylation in non-stressful conditions; (ii) the Phosphatidyl-Inositol 3-Kinase (PI3K) complex participates in the genesis of the phagophore (a pre-autophagosome structure); (iii) the ATG9 complex, which recruits portions of the ER membrane for phagophore expansion; and (iv) the ATG12 complex, which assists in cargo recruitment [14,15,16,17].

## 2. Autophagy as Part of the Vesicle Trafficking in Seeds

The origin of seeds is a decisive milestone in plant evolution. The appearance of seeds in spermatophytes allowed these plants to spread over long distances, arrest their life cycle when adverse conditions are met, and resume growth under favorable environments [18]. A seed crop is a staple food that feeds humans and animals because of its content of proteins, carbohydrates, and oils. Seed yield and vigor are relevant for modern agriculture, and depend on the seed’s nutritional value and its ability to germinate across a wide range of environmental conditions [19,20].

The accumulation of reserves during maturation and their mobilization during germination are central cellular processes that control seed yield and vigor. Seeds mainly accumulate carbohydrates (starch), proteins (Seed Storage Proteins, SSPs), and oils (triacylglycerols TAGs). Starch accumulation occurs in plastids, leading to amyloplast formation. Similarly, oil biosynthesis begins at the plastid but finishes when the ER releases oil bodies into the cytoplasm [21]. However, SSP synthesis occurs in the ER boundaries when proteins accumulate in Protein Storage Vacuoles (PSVs) and Protein Bodies (PBs)—see [22] and references therein. Protein accumulation in the ER during seed maturation resembles the aggregation of proteins that occurs during ER stress, which eventually activates autophagy to relieve protein overproduction under environmental stress conditions [23,24]. PSV formation implies the engulfment of the pre-existing Lytic Vacuole (LV) in an autophagy-like process, generating two compartments with different functions: storage (matrix and crystalloid) and lytic (globoid) [25]. This separation may assure that an initial source of hydrolytic enzymes is needed for protein degradation during early imbibition (preceding germination). SSPs require post-translational processing by proteolytic cleavage, carried out by Vacuolar Processing Enzymes (VPEs) such as cysteine proteases [26]. While PSVs store SSPs in the cotyledons, embryonic axis, and aleurone cells of both dicot and monocot seeds, PBs accumulate SSPs in the starchy endosperm of monocots [27].

**Figure 1:**
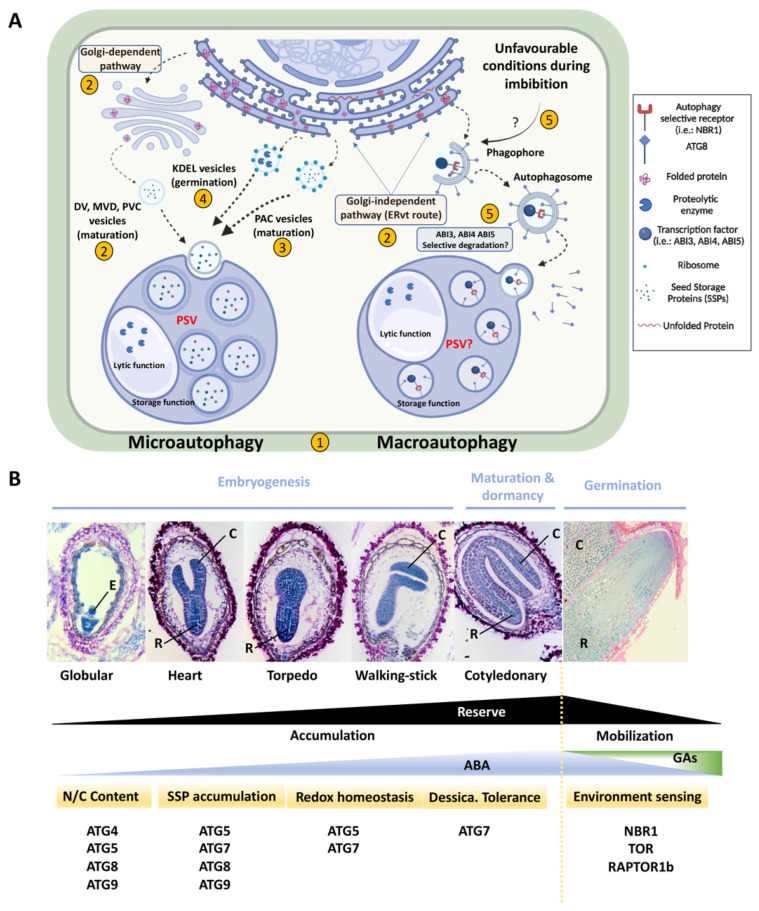
(**A**) Schematic representation of possible autophagy-like mechanisms in seeds. The Golgi-dependent pathway participates in the transport of DV, MVD, and PVC vesicles to the vacuole in a microautophagy-like mechanism during seed maturation. However, PAC and KDEL vesicles move from ER to PSV through the Golgi-independent route (also a microautophagy-like process). During seed imbibition, macroautophagy controls the expression of seed-specific transcription factors (ABI3, ABI4, and ABI5; ABA signaling) by a selective degradation mediated by ATG8 and the cargo receptor NBR1. Under unfavorable conditions, autophagy-selective degradation ceases, thus inhibiting seed germination. DV: Dense Vesicle; MVB: Multivesicular Vesicle Body; PAC: Pre-Accumulating Vesicle; PSV: Protein Storage Vacuole; PVC: Pre-Vacuolar Compartment. Picture created with BioRender.com (https://biorender.com/; accessed on 7 October 2022); numbers within yellow circles indicate sections in the manuscript where the particular process is described and references are provided. (**B**) Summary of the role of certain autophagic factors during seed development. Images illustrate a dicotyledonous seed from the globular stage during embryogenesis to germination. Sections are stained with naphthol blue-black (proteins in blue) and periodic acid plus Schiff’s reagent (insoluble polysaccharides in pink; R.I-F. contribution). ABA, GAs, and reserve levels are shown throughout seed development. ATG factors described as relevant in several aspects of seed development are indicated. C: Cotyledon; E: Embryo; R: Radicle. Mechanisms depicted in (**A**,**B**) are mainly taken from [10,27,28,29,30,31,32,33], and summarize all the content included in this mini-review.

As depicted in Figure 1, SSP transport occurs by two alternative routes: (i) a direct pathway from the ER (Golgi-independent) to PSV; and (ii) through the classical cellular transport pathway with an intermediate step at the Golgi apparatus (Golgi-dependent). The Golgi-independent route is an unconventional vesicular track known as the “ER to vacuole trafficking” (ERvt) pathway [28]. Both routes involve mechanisms similar to autophagy (Figure 1). During seed imbibition, de novo synthesis of proteolytic enzymes starts at the ER and finishes when ER-derived vesicles arrive at PSVs to mobilize SSPs. The transport of reserve proteins in seeds implies several types of intermediate vesicles: (i) Dense Vesicles (DVs), MultiVesicular Bodies (MVBs), and Pre-Vacuolar Compartments (PVCs) required for the Golgi-dependent route; and (ii) Precursor-Accumulating (PAC) vesicles involved in the Golgi-independent pathway (Figure 1) [10,19,27,28].

## 3. Autophagy in Seed Formation: Nutrient Allocation and Reserve Accumulation

Autophagy is a relevant process affecting seed quality, as disclosed by nutrient content analysis and the performance of seeds of *atg* mutants when sown or stored [34,35]. Zygotic embryogenesis, cell division, and histodifferentiation lead to the diversification into endosperm and embryonic tissues. At maturation, the seed accumulates reserves, acquires desiccation tolerance, and enters primary dormancy as a final stage [36]. Frequently, dicot seeds store reserve materials in the cotyledons, while monocot seeds do so in the endosperm. Therefore, autophagy-like processes related to reserve allocation occur in different tissues, depending on the seed type. Likewise, nutrients are mobilized through the phloem to the apoplast between the maternal and filial tissues: the seed coat in dicotyledonous seeds, and the placenta-chalazal region in monocotyledonous seeds [19]. 

During zygotic embryogenesis, autophagy is involved in nutrient allocation from senescent vegetative tissue (mainly leaves) to the growing embryo, and represents a critical step for nitrogen, sulfur, and metal remobilization [34,35,37,38,39]. Upon seed maturation, involvement of the ERvt pathway, an autophagy-like process, has been consistently reported in *Ricinus communis*, *Vigna mungo*, *Zea mays*, and several species of *Cucurbita*. It participates in the transport of SSP precursors to the cytoplasm via PAC bodies in cereal endosperm cells, and in the transport of SSPs to PSVs in aleurone cells of monocot seeds and embryo cells of dicots [27,40,41,42]. 

Global transcriptomic data in *Arabidopsis thaliana* seeds show that *ATG* genes are up-regulated, especially in the pericarp, funiculus, and chalazal maternal tissues during embryogenesis, and in the embryo during seed maturation [10,43,44]. Several genetic studies carried out in this species have demonstrated the relevance of autophagy in seed biology. Thus, the knock-out mutants *atg5*, *atg8a*, and *atg9* produce mature seeds with significant alterations in the N- and C-terminal contents, whereas the *atg7* and the double *atg4a–4b* mutants generate mature dry seeds with a lower weight than those of the wild-type. Likewise, *atg5* and *atg7* show reduced content of storage proteins such as 12S globulins and 2S albumins. Additionally, *atg5* seeds display premature development compared to the wild type, since protein storage deposition begins earlier. The stable overexpression of *AtATG5* and *AtATG7* genes improves seed production, and overexpression of the *AtATG8a* and *AtATG8g* genes results in a significant increment in the N content of seeds [10,29,30]. Concerning other storage compounds, there is evidence of autophagy involvement in lipid turnover and mobilization in yeast, algae, and animals. Notably, the fatty acid content in seeds of *Arabidopsis thaliana* plants overexpressing *ATG5* and *ATG7* is higher than that of the wild-type [45]. In addition, a recent study confirmed the direct contribution of basal autophagy to triacylglycerol (TAG) synthesis and lipid droplet degradation in leaves and seeds of Arabidopsis plants [46]. 

Interestingly, *atg5* plants contain higher levels of molecules involved in redox homeostasis (e.g., glutathione) than wild-type plants. Similarly, the level of ROS-scavenging catalase enzymes is also up-regulated in Oryza sativa *Osatg7-1* mutant seeds [47], suggesting a bond between autophagy and oxidative stress management, therefore supporting that reactive oxygen species (ROS) might affect (i) the maturation of the SSP precursors, (ii) the transport of the SSP precursors to the MVBs and/or to the PSVs, and (iii) the processing of SSP precursors into mature SSPs [30]. 

In *Oryza sativa* seeds, autophagy contributes to starch quality in the endosperm during seed maturation. The autophagy-deficient mutant *Osatg7-1* produces fewer and smaller seeds than the wild type, as occurs in the A. thaliana *atg7* mutant. Additionally, *Osatg7-1* seeds present a chalky appearance and lower starch content in the endosperm of mature seeds. These chalky seeds contain higher levels of several Heat Shock Proteins (HSPs), Late Embryogenesis Abundant (LEA) proteins, and chaperones than the wild-type ones. This suggests a link between autophagy and the acquisition of tolerance to desiccation before dormancy establishment [47].

## 4. Autophagy in Seed Germination: Reserve Mobilization

Seed germination begins with water uptake and terminates when the radicle protrudes through the layers surrounding the embryo (germination *sensu stricto*). The protrusion also denotes the starting point of post-germination events, where the hydrolysis of seed storage reserves mainly sustains seedling growth until photosynthetic capacity is acquired. During seed germination, extensive protein breakdown occurs through the action of endo- and exo-peptidases—mostly cysteine proteases, but also serine, aspartic, and metalloproteases. These enzymes are stored within PSVs as an initial battery of proteases needed to start SSP degradation during early seed imbibition and become functionally active when their zymogens undergo N-terminal peptide proteolysis [20,48].

During post-germination, significant SSP hydrolysis occurs, sustained by de novo-synthesized proteases in the cotyledons [49]. In *Arabidopsis thaliana*, the ERvt pathway transports cysteine proteases synthesized at the ER to PSVs. These cysteine proteases contain a C-terminal K/HDEL signal, characteristic of ER-resident proteins, and they are transported as inactive aggregates within KDEL Vesicles (KVs). In maize aleurone and *Vigna mungo* cotyledons, PSVs engulf KVs by a microautophagy-like process during seed germination [50,51,52]. Interestingly, starch hydrolysis during germination in *Vigna mungo* seeds also implies the engulfment of alfa-amylase enzyme by PSVs through a microautophagy-like mechanism [50]. In the *Osatg7-1* mutant, the *α-amylase 3* gene is up-regulated in germinating grains, suggesting a molecular association between starch mobilization and autophagy [47]. 

## 5. Seed Autophagy, Endoplasmic Reticulum (ER), and Environmental Responses

In Nature, seeds are immersed in the soil seedbed, which is a complex environment exposed to multiple stresses. In this context, seeds develop several molecular and cellular mechanisms to respond to environmental fluctuations and guarantee offspring survival [20]. In eukaryotes, the endoplasmic reticulum (ER) is the central cellular organelle regulating stress responses [53]. In the seedbed ecosystem, activation of different stress responses leads to the accumulation of an excess of proteins in the endoplasmic reticulum (ER stress). Cell homeostasis is restored by the Unfolded Protein Response (UPR) pathway [53], which helps reduce ER-accumulated unfolded proteins. When ER stress persists, autophagy is activated as a feedback mechanism to alleviate protein overaccumulation [54]. The specific removal of certain ER domains via autophagy (ER-phagy) has been described as a central mechanism for maintaining cell homeostasis [55]. In *Arabidopsis thaliana*, the Inositol-Requiring 1 (IRE1) enzyme, an ER transmembrane protein with kinase and nuclease activities, has been involved in the degradation of RNAs to prevent the translation of proteins that interfere with autophagy. Accordingly, *ire1* mutants are more sensitive to different environmental stresses [56,57,58]. Although there is no evidence for a similar role for IRE1 in seed germination in response to stress conditions, in *Medicago truncatula*, the model plant for Leguminosae, autophagy and ER-stress are implicated in seed development and drought stress responses through the participation of *MtATGs* [59].

Autophagy has classically been considered a non-selective bulk degradation mechanism, but recently, the specific degradation of proteins (e.g., transcription factors, TFs), lipid droplets, and organelles (i.e., mitochondria, peroxisomes, etc.) has been related to responses to several biotic and abiotic stresses, including nutrient starvation or salt stress [32,60,61,62]. In addition, genetic studies in *Arabidopsis thaliana* have shed light on its potential implications for the seed. The knock-out mutant of *Regulatory-Associated Protein of Tor1b* gene (*raptor-1b*—a TOR interactor) produces seeds with delayed germination, reduced resistance to stresses, low viability, and high concentrations of the phytohormone abscisic acid (ABA), a well-known inhibitor of seed germination. Interestingly, when the phytohormone gibberellic acid (GA) is added to the *raptor-1b* seeds, they recover normal germination. Moreover, *raptor-1b* seeds germinate more slowly than wild-type seeds when undergoing accelerated aging, osmotic stress, and salt stress. All these data suggest that TOR acts as an “autophagy switcher” and is a major player in controlling seed germination in response to environmental changes [63].

The autophagy factor ATG8 (a ubiquitin-fold protein) holds an outstanding position in the selective recruitment of autophagy cargos. ATG8 interacts with other specific receptor proteins (e.g., Neighbor of *BRCA1* gene 1, NBR1) that contain ATG8-interacting motifs (AIMs) that recognize autophagy tags [3,15]. In *Arabidopsis thaliana*, *Solanum lycopersicum*, and *Zea mays*, several autophagy receptors have been described that interact with ATG8 through AIMs, and transport cargos from the ER to the vacuole (ATI1/2, ATI3, Sec62, Rtn, TSPO, etc.; reviewed in [31]). Arabidopsis plants overexpressing AtNBR1 produce seeds that are less sensitive to ABA than wild-type seeds when ABA is added to the imbibition medium [32,33]. Similarly, *Triticum aestivum* transgenic plants overexpressing the *TaNBR1* gene show lower seed germination rates under drought stress conditions than wild-type seeds, suggesting a role for TaNBR1 in response to drought stress during germination [64]. Interestingly, the *Arabidopsis thaliana* NBR1 receptor has been reported to be essential for fine-tuning ABA signaling by physically interacting with transcription factors ABI3 (B3-subfamily, ortholog to VP1), ABI4 (ERF/AP2), and ABI5 (bZIP-family). ABI3, ABI4, and ABI5 are involved in ABA signaling and the control of seed dormancy, preharvest sprouting (PHS), and germination in orthodox seeds. PHS is one of the most significant seed quality defects, causing important economic annual losses of approximately USD 1 billion worldwide [31,65,66].

## 6. Concluding Remarks and Future Perspectives

Seeds are the most important world crop, and seed yield and germination performance in response to environmental fluctuations are two key features of modern agriculture. This review compiles recent advances supporting the relevance of autophagy in these crucial seed parameters. Autophagy-like mechanisms have been described as essential at the cellular level for reserve accumulation and mobilization during seed development. Genetic studies have also demonstrated the importance of several ATG factors in the regular course of seed development.

One of the most exciting aspects concerning the impact of autophagy on the seed is how it can modulate developmental processes and responses to the environment by controlling the selective degradation of transcription factors and other molecular switches. Tarnowski et al. [33] have demonstrated such a mechanism for ABI3 (ortholog to VP1), ABI4, and ABI5; the major transcription factors controlling ABA sensing during seed dormancy and germination [31,66] (Figure 1). These results are the basis for subsequent studies to answer important questions concerning (i) the involvement of autophagy in the seed as a general response mechanism to environmental changes and (ii) the identification and characterization of selective autophagy receptors that participate specifically in the seed (TSPO, RPN10, etc.) [31]. Major advances in the genetics and molecular basis of seed autophagy will be essential to the development of new plant varieties by targeting autophagy genes with high seed yield and germination vigor in response to changes in the ecosystem.
